# Genome Maintenance by DNA Helicase B

**DOI:** 10.3390/genes11050578

**Published:** 2020-05-21

**Authors:** Lindsey Hazeslip, Maroof Khan Zafar, Muhammad Zain Chauhan, Alicia K. Byrd

**Affiliations:** 1 Department of Biochemistry and Molecular Biology, University of Arkansas for Medical Sciences, Little Rock, AR 72205, USA; lhazeslip@uams.edu (L.H.); mkzafar@uams.edu (M.K.Z.); mzchauhan@uams.edu (M.Z.C.); 2 Winthrop P. Rockefeller Cancer Institute, Little Rock, AR 72205, USA

**Keywords:** DNA replication, DNA repair, DNA damage, genomic stability, DNA helicase

## Abstract

DNA Helicase B (HELB) is a conserved helicase in higher eukaryotes with roles in the initiation of DNA replication and in the DNA damage and replication stress responses. HELB is a predominately nuclear protein in G_1_ phase where it is involved in initiation of DNA replication through interactions with DNA topoisomerase 2-binding protein 1 (TOPBP1), cell division control protein 45 (CDC45), and DNA polymerase α-primase. HELB also inhibits homologous recombination by reducing long-range end resection. After phosphorylation by cyclin-dependent kinase 2 (CDK2) at the G_1_ to S transition, HELB is predominately localized to the cytosol. However, this cytosolic localization in S phase is not exclusive. HELB has been reported to localize to chromatin in response to replication stress and to localize to the common fragile sites 16D (FRA16D) and 3B (FRA3B) and the rare fragile site XA (FRAXA) in S phase. In addition, HELB is phosphorylated in response to ionizing radiation and has been shown to localize to chromatin in response to various types of DNA damage, suggesting it has a role in the DNA damage response.

## 1. Introduction

Helicases are vital to any event that requires the separation of the two strands of DNA or RNA such as DNA repair, replication and recombination. Several helicases in the RecQ and iron-sulfur helicase families are known to be essential for maintaining genomic stability [1]. Genetic defects associated with these helicases cause premature aging and predisposition to cancer. DNA Helicase B (HELB, DHB, or HDHB) is a superfamily 1B helicase that also has roles in genome maintenance. HELB is highly conserved among vertebrates but has no known orthologs in lower eukaryotes [2]. The gene for murine HELB showed similarities to the *Bacillus subtilis* RecD2 and *E. coli* RecD [3]. Preliminary studies with mouse and human HELB showed it hydrolyzes ATP and unwinds DNA in the 5′-3′ direction; however, a detailed biochemical analysis is lacking [2,4]. A heat sensitive mutant of HELB was first discovered in murine FM3A cells [4]. When these cells were arrested in early S phase, HELB expression in the nucleus was increased [3]. This mutant became inactive at increased temperatures, and the cells with inactive HELB showed a decreased incidence of DNA replication compared to wild type cells although the rate of elongation was unaffected [4]. This suggests that the helicase functions primarily in the early stages of S phase. Mouse HELB co-purified with DNA primase and stimulated synthesis of short primers but not long oligonucleotides by DNA primase [5], suggesting a role for mouse HELB in initiation of DNA synthesis. However, after treatment with hydroxyurea to deplete the dNTP pools, the replication rate in HELB knockout mouse embryonic fibroblasts dropped, thus suggesting a role for mouse HELB in the recovery from replication stress [6]. HELB knockout mice are normal under unchallenged conditions [6], and the effects of endogenous replication stress on these mice are still unknown.

## 2. Domain Structure

**Figure 1:**
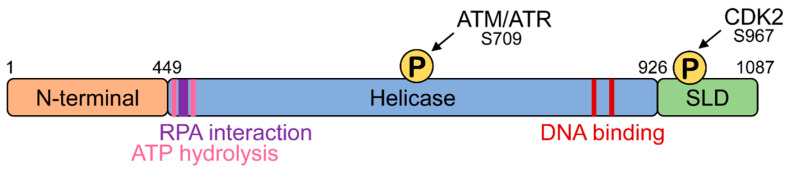
HELB domain structure. HELB has a N-terminal domain, a helicase domain that binds DNA [6], hydrolyzes ATP [2], and interacts with RPA [7], and a subcellular localization domain (SLD) [7]. The SLD is phosphorylated by CDK2 at the G_1_ to S transition [7] and the helicase domain is phosphorylated in response to ionizing radiation [12]. Note that the boundary between the N-terminal domain and helicase domain here is different than originally reported [2] due to the discovery of the Q-motif N-terminal to the first helicase motif identified at the time of the original report [9,13].

Human HELB is 1087 amino acids long and contains three functional domains: an amino terminal domain, a central helicase domain, and a carboxy terminal domain (Figure 1) [7]. Although the function of the N-terminal domain is not completely understood, it has been shown to physically interact with CDC45, a component of the CMG (CDC45, MCM2–7, GINS) replicative helicase, in vitro [8], suggesting that the N-terminal domain may function in protein–protein interactions. The helicase domain contains the 11 conserved motifs of the Pif1/RecD2-like family of superfamily 1 helicases [9]. The helicase domain contains a site located in an acidic motif (residues 493–517) between the Walker A (residues 475–482) and Walker B (residues 590–594) helicase motifs involved in ATP hydrolysis that interacts with the single-stranded DNA-binding protein RPA [10]. In addition to interacting with the N-terminal domain, CDC45 also associates with the helicase domain in vitro [8]. The helicase domain also contains an ATM/ATR phosphorylation site at serine 709. The carboxy terminal subcellular localization domain contains a cyclin-dependent kinase phosphorylation site [7], a nuclear localization sequence [10,11], and a nuclear export sequence [7].

## 3. Subcellular Localization

The localization of human HELB is cell cycle dependent. Subcellular fractionation followed by immunoblotting and fluorescence microscopy showed that HELB localizes to both the nucleus and cytoplasm in asynchronous and unstressed cells [7]. However, in G_1_ phase, HELB is predominantly nuclear. Phosphorylation of S967 in the SLD domain by CDK2 during the late G_1_ phase results in the export of the majority of HELB to the cytoplasm during S phase [7], although some HELB remains in the soluble nuclear fraction [10]. Both cyclin E/CDK2 and cyclin A/CDK2 were able to phosphorylate HELB in vitro, but it was suggested that, due to the co-immunoprecipitation of cyclin E with HELB, cyclin E/CDK2 is the complex which phosphorylates HELB, targeting it for nuclear export [7]. However, cyclin A2 also associates with HELB [6], suggesting that either cyclin E/CDK2 or cyclin A2/CDK2 could be responsible for the phosphorylation of the HELB at S967. The CDK2-dependent re-localization of HELB suggests that HELB may have different roles depending on the phase of the cell cycle which need to be explored further.

## 4. Functions of Human HELB

### 4.1. Role in DNA Replication

**Figure 2:**
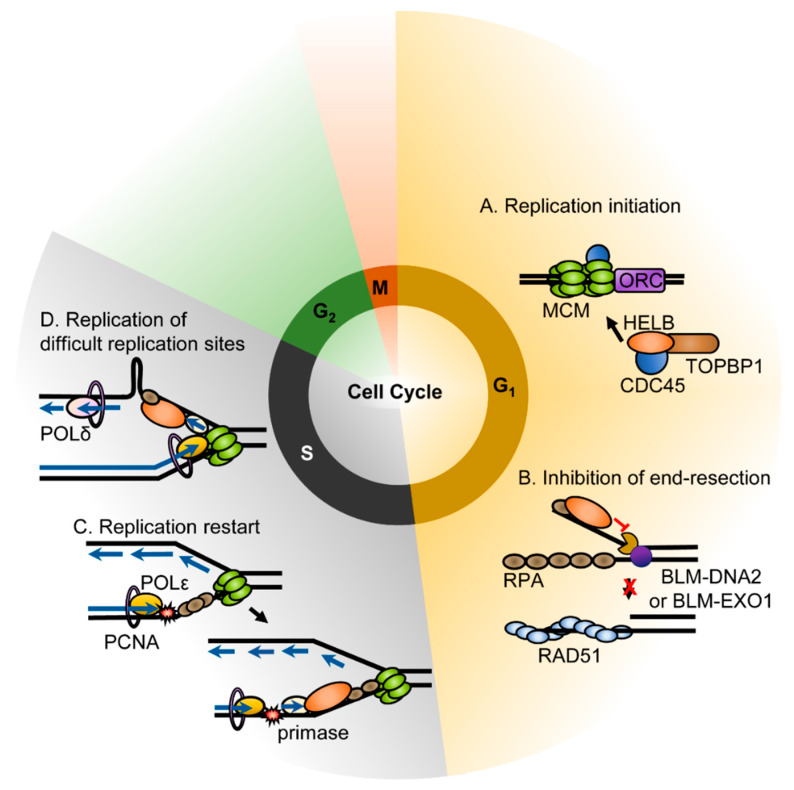
Functions of HELB. Roles for HELB in loading the pre-initiation complex (**A**), inhibition of end resection (**B**), recovery from replication stress (**C**), and in replication of fragile sties (**D**) have been proposed [2,6,10,15].

Similar to mouse HELB, recombinant human HELB also interacts with DNA polymerase α-primase (pol-prim) and stimulates the synthesis of RNA primers [2]. HELB also overcomes the inhibition of RPA on pol-prim mediated RNA primer synthesis [2]. Due to the interaction of HELB with pol-prim, ATPase-deficient HELB variants have a dominant negative phenotype. HeLa cells microinjected with wild-type HELB in G_1_ phase progressed into S phase normally, whereas cells micro-injected with HELB containing mutations in the Walker A or B motifs exhibited delayed entry into the S phase, indicating that HELB is required for timely cell cycle progression [2]. However, normal DNA replication proceeded if HELB variants were injected after cells entered S phase [2]. In addition, transient knockdown of HELB in U2OS cells reduced BrdU incorporation into newly synthesized DNA and caused cells to arrest in G_1_ phase [8]. This indicates that HELB may be required for replication initiation during the G_1_ phase (Figure 2A). Moreover, the Fanning lab showed that HELB interacts directly with both CDC45 and TOPBP1 [8] and depletion of HELB disrupts the initiation of replication prior to the stable loading of CDC45 on chromatin due to both a decrease in CDC45 recruitment to chromatin and a delay of S phase [8]. CDC45 and TOPBP1 are components of the pre-initiation complex [14]. The association of HELB with these components suggests that the helicase plays a role in the assembly of the pre-initiation complex. HELB localizes to replication origins during G_1_ [8], suggesting that it may unwind the origin to load the pre-initiation complex.

The functional role of HELB in ongoing DNA replication is not fully understood. Other than the lack of an effect of HELB ATPase deficient variants that were injected during the S phase [2], we are not aware of any studies of the function of HELB during replication progression. However, HELB has been shown to localize to fragile sites during S phase. The helicase was enriched at the repeat region of the FRAXA rare fragile site in the *FMR1* gene, the FRA16D common fragile site (CFS) in the *WWOX* gene, and the FRA3B CFS in the *FHIT* gene [15]. The CGG repeats at FRAXA can form both hairpin and G-quadruplex structures that stall replication in vitro and in vivo [16,17,18,19]. HELB may be recruited to these regions to unwind secondary repeat structures ahead of DNA polymerase (Figure 2D). The AT-rich CFS such as FRA16D and FRA3B have also been reported to form secondary structures in vitro that align with polymerase stall sites [20,21] suggesting that HELB may also unwind secondary structures at CFS. However, other models of CFS instability such as collisions of replication and transcription complexes [22], formation of R-loops during transcription [23,24], and a paucity of origins in the vicinity of CFS [25] have all been proposed, making the exact role of HELB at these sites difficult to predict.

### 4.2. Response to Replication Stress

Depletion of HELB in HeLa cells leads to a modest increase in checkpoint signaling as phosphorylation of CHK1 and phosphorylation of RPA at S4 and S8 increase after treatment with hydroxyurea [10]. Cells lacking HELB exhibit impaired recovery from replication stress. Cell survival decreased and chromosomal aberrations increased in the absence of HELB after replication stress, suggesting that HELB promotes the resolution of replication stress [10].

Replication stress such as the stalling of the replication fork leads to long stretches of ssDNA coated with RPA. Several studies have shown that HELB interacts with RPA. Two separate groups performed proteomic screens to identify RPA interacting proteins, one using nuclear extracts of HEK-293T cells, and the other using whole cell extracts of HEK-293 cells; both identified HELB as an RPA70-interacting protein [6,26]. The Fanning lab also identified a conserved acidic motif between the Walker A and B motifs (Figure 1) that directly interacts with a basic cleft of the RPA70 N-terminal OB-fold domain [10]. Isothermal titration calorimetry with the acidic peptide from HELB and the RPA70 N-terminal domain revealed affinity in the low micromolar range [10]. Furthermore, NMR and immunoprecipitation experiments confirmed the physical interaction between HELB and RPA70 and identified acidic residues E499, D506 and D510 as vital to this interaction [10].

HELB is recruited to chromatin by RPA, but not upon checkpoint signaling, in the response to replication stress [10]. HELB recruitment to chromatin correlates with the level of chromatin-bound RPA [10]. HELB localizes to chromatin in response to the treatment of cells with etoposide, camptothecin, hydroxyurea, UV radiation, and ionizing radiation [7,10] implying a role in the DNA damage response. During S phase, chromatin bound HELB increases through re-localization of the soluble nuclear HELB, but not through import of the cytosolic HELB [10]. It remains unknown how the recruitment of HELB to the chromatin and the interaction with RPA are involved in the recovery from replication stress. It has been suggested that HELB may work to re-prime the leading strand downstream of stalled replication forks based on its interaction with pol-prim [10] (Figure 2C). However, re-priming the leading strand in higher eukaryotes likely involves DNA-directed primase/polymerase protein (PRIMPOL) instead of pol-prim [27]. PRIMPOL is a DNA primase and DNA polymerase in higher eukaryotes that is involved in re-priming downstream of stalled replication forks and in translation synthesis [27]. Pol-prim is RNA primase and DNA polymerase that synthesizes RNA primers with a short DNA extension during initiation of DNA synthesis [28]. It is not known whether HELB also interacts with PRIMPOL. Alternatively, HELB may be able to promote the resolution of a reversed fork in a manner similar to RECQ1 helicase [29]. The exact mechanism of HELB in the replication stress response is unknown and will be important to determine in the future.

### 4.3. Response to DNA Damage

The DNA damage response pathway is critical for maintaining genomic integrity. Double-strand breaks (DSBs), which are particularly deleterious, are repaired by either nonhomologous end joining (NHEJ) or homologous recombination (HR). Although NHEJ is more error prone than HR, DSBs are only repaired via HR when there is a sister chromatid present as a repair template. When there is no sister chromatid, the damage is repaired with NHEJ. In order for HR to occur, the ends of DNA must be resected to generate the 3′-overhangs needed for strand invasion. Once end resection has occurred, the cells are committed to repairing the break by HR [30]. For this reason, initiation of end resection is highly regulated. HELB is central to one of the mechanisms regulating end resection. End resection occurs in two steps. First, short range resection by MRE11 in complex with RAD50 and NBS1 (the MRN complex) occurs after initiation by the CtIP endonuclease. This is followed by long range resection by the 5′-3′ exonuclease EXO1 and DNA2 which has 5′-3′ helicase and endonuclease activity [30]. After initiation of resection, the ssDNA is coated with RPA. HELB is recruited to DSBs by RPA. This is dependent on the acidic RPA interaction motif in HELB but is independent of its catalytic activity [6]. HELB then inhibits a long-range resection by EXO1 and BLM-DNA2 [6] (Figure 2B). The mechanism of inhibition of resection is unknown; however, based on the ability of HELB to displace proteins from DNA, it has been proposed that HELB uses its ATPase activity to dissociate BLM-DNA2 or EXO1 from the DNA after being recruited by RPA [6]. The 5′-3′ directionality of HELB suggests that HELB may bind the 5′-tail resulting from unwinding by BLM instead of the 3′-tail where it would translocate away from the nucleases [31]. In order to suppress long-range end resection, HELB requires ssDNA binding, RPA interaction, and catalytic activity [6]. This inhibition is independent of 53BP1 suppression of end resection; loss of both 53BP1 and HELB results in an additive increase in end resection, indicating they function in separate pathways. HELB also does not directly affect repair by NHEJ [6]. HELB localizes to the nucleus in G_1_ phase and the cytosol in S and G_2_ phases, thus resulting in an increase in long-range end resection during the S phase.

BRCA1- and BRCA2-deficient tumor cells are sensitive to PARP inhibitors (PARPi), due, in part, to their inability to repair DSBs by HR [32]. However, several mechanisms of PARPi resistance have been reported [32]. The most common mechanisms of drug resistance involve acquisition of new mutations which result in a functional BRCA protein or restoration of HR by loss of 53BP1 [33,34,35]. Knockdown of HELB in BRCA1-deficient mammary tumor cells results in resistance to PARPi, which suggests that BRCA1-independent HR is activated in the absence of HELB [6,36]. This is likely due to an increase in end resection in the absence of HELB [6]. Since HELB DNA-binding motifs, RPA interaction motif, and catalytic activity are required to suppress end resection [6], this suggests that, similar to loss of 53BP1 activity [33,34,35], mutations in any of these critical regions of HELB would render BRCA1-deficient tumors resistant to PARPi. This idea needs to be investigated in tumor samples.

## 5. Regulation

### 5.1. Transcriptional Regulation

At the level of transcription, *HELB* expression is controlled by transcription factors such as STAT-x, Sp1 and c-ETS [37]. Interestingly, transcription of *HELB* increases in the response to resveratrol [37]. This is modulated by the GC-box/Sp1 binding sites and the duplicated GGAA-motif in the c-ETS binding site suggesting that either Sp1 or c-ETS may be involved. Resveratrol is an estrogen receptor agonist [38], and the estrogen receptor can modulate binding of Sp1 [39], suggesting that Sp1 may be responsible for the changes in *HELB* expression with resveratrol. This also suggests that *HELB* transcription may respond to estrogen.

The region of the *HELB* promoter containing the GC-boxes/Sp1 binding sites also has the potential to fold into G-quadruplex or i-motif structures. A recent bioinformatic analysis found sequences with the potential to form G-quadruplexes in the promoters and 5′-UTRs of several DNA repair genes including *HELB* [40], and the opposite strand has the potential to form i-motifs. G-quadruplexes and i-motifs are four stranded structures that can form in G- or C-rich regions of the genome, respectively, that have been shown to regulate transcription of multiple genes [41,42,43,44]. The presence of putative G-quadruplex and i-motif sequences in the *HELB* promoter suggests that *HELB* transcription may also be regulated by formation of these structures although this needs to be tested empirically.

### 5.2. Post-Translational Regulation

As described earlier, HELB is also regulated by phosphorylation by CDK2 [7,45]. HELB is predominately a nuclear protein in G_1_ phase [7]; after phosphorylation by CDK2 at the G_1_ to S transition, HELB is predominately localized to the cytosol [7]. Mutations in the HELB nuclear export sequence increase its nuclear localization during S and G_2_ phases [6]. This re-localization of the majority of HELB to the cytosol for S phase appears to serve two important purposes. First, it may prevent re-replication of the DNA since HELB functions in the assembly of the pre-initiation complex [8]. Second, it would relieve the inhibition on long-range end resection to allow DSB repair to proceed by homologous recombination after sister chromatids are synthesized [6].

In addition to the cell cycle-dependent phosphorylation, HELB is phosphorylated in response to ionizing radiation on an ATM/ATR consensus site [12]. ATM and ATR kinases are activated by DNA damage and replication stress, respectively, and they phosphorylate effector proteins to signal DNA damage and activate the DNA damage response [46]. The specific role of HELB in the DNA damage response and replication stress response are unknown, but HELB negatively regulates long-range end resection in G_1_ phase [6] and localizes to chromatin in response to DNA damage in the S phase [10]. The molecular functions of HELB in response to replication stress remain to be determined.

HELB phosphorylation has also been detected in phospho-proteomic screens at several additional sites, most often in the C-terminal SLD [47]. Phosphorylation at various sites was detected in normal and cancer cells, with and without treatment with drugs and inhibitors of different signaling pathways. Inference of the functional role of these phosphorylation events is difficult based on the existing data and will require further study.

The cell cycle-dependent regulation of HELB is not entirely dependent on cyclin-dependent kinases and the nuclear export sequence, as some cell cycle-dependent regulation is still observed when the nuclear export sequence is mutated [6]. HELB may also be regulated by ubiquitin attachment. Ubiquitylation of multiple sites on HELB has been detected in proteomics screens using antibodies to detect ubiquitin modifications [48,49,50]. These modifications may regulate HELB levels by ubiquitin-dependent proteolysis. Supporting this idea is the observation that HELB interacts with the E3 ubiquitin ligase SKP2 [6]. In addition, many proteins involved in the replication stress response are ubiquitylated when the replication fork stalls to initiate DNA repair [51]. Hence, ubiquitylation of HELB may activate its role in the replication stress response. The role of each of these ubiquitin modifications remains to be determined.

## 6. Effects of Variants

Interestingly, two low frequency, missense variants of HELB (rs75770066 and rs148126992) are associated with the premature onset of natural menopause [52]. In fact, these two single-nucleotide polymorphisms (SNPs) in high linkage disequilibrium were the only signal which reached genome-wide significance in the discovery phase [52]. A younger age at natural menopause is correlated with a greater risk of osteoporosis and heart disease and a decreased risk of breast cancer [53,54,55]. Age at menopause also affects fertility since fertility often ends 10 years before menopause [56]. Like age at natural menopause, a cluster of single-nucleotide polymorphisms (SNPs) in *HELB* is also associated with female infertility, based on data from the Michigan Genomics Initiative (MGI) (Table 1).

DSB repair associated with meiotic recombination has a major effect on oocyte quantity and, therefore, age at natural menopause [52]. Meiotic recombination is similar to homologous recombination in many ways and relies on the same end resection machinery [57]. Aberrant meiotic recombination results in cell cycle arrest and reduced oocyte viability, as incorrectly repaired DSBs can easily lead to genome instability and trigger apoptosis [52]. Interestingly, one of the HELB variants associated with age at natural menopause (rs75770066) is D506G. Aspartate-506 (D506) is in the acidic peptide that interacts with RPA [10], suggesting that impaired interactions with RPA may be the cause of this phenotype. The effect of this amino acid change alone is unknown, but a combination of E499A, D506A, and D510A in vitro is sufficient to interfere with the localization to DNA in response to replication stress [10] and relieve the inhibition on end resection [6]. Since HELB negatively regulates homologous recombination through its interaction with RPA, this suggests HELB may also limit meiotic recombination in an RPA dependent manner. This would prevent excessive recombination and suggests that HELB’s role in DSB repair may be critical in oocytes.

## 7. Conclusions

HELB has multiple functions in DNA replication and repair. However, many questions remain to be answered about HELB’s role in these processes as many of the molecular details are unknown. In particular, little is known about how HELB increases a cell’s ability to withstand replication stress beyond its localization to the DNA in response to treatment with agents which induce replication stress. The role of HELB in replication initiation is also not completely understood as it interacts with multiple proteins involved in this process. Although the function of HELB in response to DSBs has been well characterized, the effect of this on processes such as meiosis is unclear. However, it is clear that HELB plays critical roles in multiple processes essential to genome maintenance and its activity needs to be further studied.

## Figures and Tables

**Table 1:** HELB SNPs are Associated with Infertility in Females. Thirteen SNPs in the HELB Gene are Associated with Female Infertility Based on Data from MGI. MAF is Minor Allele Frequency; Positions were Determined Using GRCh37.

SNP	Alleles	Variant	MAF	Position (Chr:12)	P-Value
rs12301608	C/T	Intronic	0.0125	66,707,644	2.24 × 10^−5^
rs12228262	G/C	Intronic	0.0125	66,708,507	2.28 × 10^−5^
rs10878404	C/T	Intronic	0.0122	66,709,488	1.74 × 10^−5^
rs76187362	A/G	Intronic	0.0125	66,711,895	2.47 × 10^−5^
rs79976130	C/T	Intronic	0.0125	66,712,652	2.52 × 10^−5^
rs10878406	T/C	Intronic	0.0122	66,713,978	3.63 × 10^−5^
rs10878407	C/T	Intronic	0.0124	66,717,202	5.38 × 10^−5^
rs35536133	T/A	Exonic/synonymous	0.0124	66,717,784	6.06 × 10^−5^
rs28551050	G/T	Intronic	0.0124	66,718,207	6.62 × 10^−5^
rs10878408	C/G	Intronic	0.0122	66,718,973	6.31 × 10^−5^
rs139815108	C/T	Intronic	0.0113	66,718,957	3.44 × 10^−5^
rs34109029	G/T	Intronic	0.0124	66,717,910	6.29 × 10^−5^
rs60549090	G/T	Intronic	0.0122	66,705,808	1.63 × 10^−5^
